# Free-Energy Profile Analysis of the Catalytic Reaction of Glycinamide Ribonucleotide Synthetase

**DOI:** 10.3390/life12020281

**Published:** 2022-02-14

**Authors:** Norifumi Yamamoto, Genichi Sampei, Gota Kawai

**Affiliations:** 1 Department of Applied Chemistry, Faculty of Engineering, Chiba Institute of Technology, 2-17-1 Tsudanuma, Narashino 275-0016, Chiba, Japan; 2 Department of Applied Physics and Chemistry, Faculty of Electro-Communications, The University of Electro-Communications, 1-5-1 Chofugaoka, Chofu 182-8585, Tokyo, Japan; gsampei@uec.ac.jp; 3 Department of Life Science, Faculty of Advanced Engineering, Chiba Institute of Technology, 2-17-1 Tsudanuma, Narashino 275-0016, Chiba, Japan; gota.kawai@p.chibakoudai.jp

**Keywords:** QM/MM hybrid model, free-energy profile, minimum energy path, purine nucleotide biosynthesis, ATP-grasp enzyme

## Abstract

The second step in the de novo biosynthetic pathway of purine is catalyzed by PurD, which consumes an ATP molecule to produce glycinamide ribonucleotide (GAR) from glycine and phosphoribosylamine (PRA). PurD initially reacts with ATP to produce an intermediate, glycyl-phosphate, which then reacts with PRA to produce GAR. The structure of the glycyl-phosphate intermediate bound to PurD has not been determined. Therefore, the detailed reaction mechanism at the molecular level is unclear. Here, we developed a computational protocol to analyze the free-energy profile for the glycine phosphorylation process catalyzed by PurD, which examines the free-energy change along a minimum energy path based on a perturbation method combined with the quantum mechanics and molecular mechanics hybrid model. Further analysis revealed that during the formation of glycyl-phosphate, the partial atomic charge distribution within the substrate molecules was not localized according to the formal charges, but was delocalized overall, which contributed significantly to the interaction with the charged amino acid residues in the ATP-grasp domain of PurD.

## 1. Introduction

De novo purine nucleotide biosynthesis pathway is evolutionarily conserved in all organisms, including plants [1,2], microorganisms [3], and mammals [4]. Purine nucleotide synthesis starts with the formation of 5-phosphoribosyl-1-pyrophosphate from simple molecules such as carbonates, amino acids, and tetrahydrofolate, which yields inosine monophosphate (IMP) as an intermediate precursor. After IMP is produced, the pathway splits into two branches to produce adenosine monophosphate (AMP) and guanosine monophosphate (GMP). AMP and GMP are eventually phosphorylated to form adenosine triphosphate (ATP) and guanosine triphosphate.

**Figure 1:**
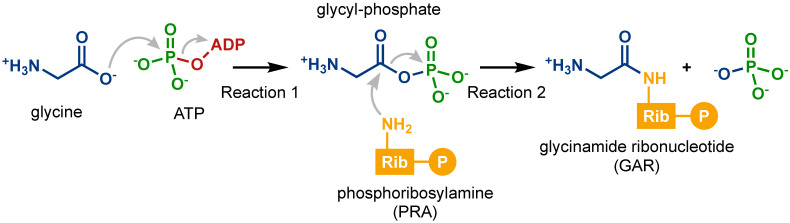
The second step in the de novo biosynthesis of purine nucleotides catalyzed by PurD. The process produces glycinamide ribonucleotide (GAR) from glycine and phosphoribosylamine (PRA) with the consumption of an ATP molecule.

The *purD*-encoded glycinamide ribonucleotide (GAR) synthetase, also known as PurD, catalyzes the second step of the de novo synthesis of purine nucleotides, which yields GAR from phosphoribosylamine (PRA) and glycine by consuming an ATP molecule [5,6,7]. This reaction consists of two steps, as shown in Figure 1. First, glycine reacts with ATP to generate a glycyl-phosphate intermediate, which is activated for nucleophilic attack. Then, glycyl-phosphate reacts with PRA, forming a carbon-nitrogen bond to yield GAR.

In addition to PurD, six more enzymes involved in the subsequent steps of the purine nucleotide biosynthetic pathway of prokaryotes are also dependent on ATP. They are PurT, PurL, PurM, PurK, PurC, and PurM, where the name of each protein is derived from its gene name [8]. PurD, PurK, PurT, and PurP show high structural similarity and are classified into the ATP-grasp superfamily of proteins, which share a unique ATP-binding site, called ATP-grasp fold, despite dissimilarities in their amino acid sequences [9]. These four enzymes catalyze the coupling of amino and carboxylate groups of the substrates, where the use of an acylphosphate intermediate is a common feature. PurD is found in all organisms. However, PurK, PurT, and PurP are found only in prokaryotes. PurD, PurK, and PurT consist of three domains labeled N, ATP-grasp, and C. The ATP-grasp domain is further divided into three subdomains labeled A, B’, and B [7]. Although the substrates are different for each of these enzymes, these ATP-grasp enzymes might share a common mechanism to catalyze the coupling between an amino group of one substrate and a carboxylate group of the other, which includes ATP cleavage, formation of an acylphosphate intermediate, and nucleophilic attack by the amino group.

The similarities in structure and catalytic strategies among the ATP-grasp enzymes of the purine nucleotide biosynthesis pathway suggest an evolutionary relationship between them. These enzymes evolved from a common ancestral ligase that can catalyze multiple ATP-dependent steps on several substrates [7,8]. Over time, each enzyme evolved to acquire a new function with a higher catalytic activity in a specific step. Enzymes with promiscuous catalytic activities and broad substrate specificities have been an interesting topic of molecular evolution [10,11]. It has been suggested that enzymes showing high specific catalytic activities divergently evolved from a progenitor enzyme with broad substrate specificities and low catalytic activities. These facts make the ATP-grasp enzymes attractive targets for understanding the molecular evolution of the purine nucleotide biosynthetic pathway [12,13]. To elucidate the evolutionary process of the purine metabolic pathway from the functional relationship of the ATP-grasp superfamily enzymes, knowledge of the reaction process at the molecular level is necessary. However, the details are unclear. Additionally, despite the importance of the acylphosphate intermediate in these reactions, it has not yet been trapped in an enzyme crystal structure.

In this study, we analyzed the molecular mechanism of the glycine phosphorylation process, Reaction 1 shown in Figure 1, the elementary process catalyzed by PurD in the de novo biosynthetic pathway of purine nucleotides. The reason why we focused only on Reaction 1 is that the binding structure of PRA to PurD, which is necessary for the analysis of Reaction 2, has not yet been clarified. The minimum energy pathway (MEP) and free-energy profile were determined based on multi-scale models using the quantum mechanical and molecular mechanical (QM/MM) method, which can quantitatively handle the large systems necessary for biomolecular modeling [14]. The focus of this study is PurD because this enzyme is a typical member of the ATP-grasp superfamily and is associated with purine nucleotide biosynthesis in all organisms. The theoretical insights provided by this study can also be useful in understanding the reaction mechanisms of other ATP-grasp superfamily enzymes, such as PurK and PurT. In this study, our results provide insights not only from the energetic aspect based on the free-energy profile but also from the viewpoint of the interactions between the substrate molecules and the enzyme during the reaction. Analysis of PurD is thus a starting point for understanding the functional relationship among the ATP-grasp superfamily enzymes, which gives a molecular-level insight into the evolutionary process of the de novo biosynthetic pathway of purine nucleotides.

## 2. Materials and Methods

### 2.1. Initial Structure

The three-dimensional structure of PurD has been determined in several organisms. In this study, we investigated the catalytic activity of PurD from *Aquifex aeolicus*, a thermophilic bacterium that is thought to be one of the oldest species of bacteria. We focused on the phosphorylation of glycine, as shown in Figure 1, which is the first step of the GAR biosynthetic reaction to produce the glycyl-phosphate intermediate. PurD binds to four substrate molecules involved in the reaction: ATP, two Mg^2+^ ions, and glycine. The initial structure of PurD was obtained from the X-ray crystal structure of *Aquifex aeolicus* PurD in an ATP-bound form (PDB ID: 2YW2) [7], after the removal of the crystallographic water molecules. Two crystal structures (PDB IDs: 2YW2 and 2YYA) of *Aquifex aeolicus* PurD have been deposited in PDB by two of the authors. In this study, we selected the ATP-bound structure, 2YW2, as the initial structure. No structure that is exactly equivalent to the initial and final states of the reaction has been identified. However, a few structures with parts of the substrates bound to PurD have been determined. Thus, plausible structures of the PurD complex with substrates for the initial and final states of the reaction were prepared according to the analyses reported by Sampei et al. [7].

### 2.2. QM/MM Hybrid Model

The hybrid model combining the QM and MM models of the QM/MM theory has been utilized as one of the most efficient tools for analyzing chemical reactions in large molecular systems such as enzyme reactions [14]. *N*-layered integrated molecular orbital molecular mechanics (ONIOM) is a type of QM/MM methods [15,16]. The QM/MM-ONIOM method was used to compute the energy and force of the system, where the total system was divided into active and environmental parts. In this study, 55 atoms in the active part, including all substrate molecules of ATP^4−^, two Mg^2+^ ions, and glycine form a QM sub-system, which is described at the density functional theory level of calculation with the B3LYP functional and the 6-31G(d,p) basis sets. The remaining 6717 atoms in the environment part modeling the enzyme structure formed an MM sub-system, which was described by the AMBER parm96 force field [17]. The QM and MM subsystems in the model are not combined with covalent bonding. However, they interact with each other through van der Waals and electrostatic interactions. The electron embedding method was used to quantitatively determine the electrostatic term in the intermolecular interaction since the partial charges on atoms in the QM-subsystem can change during the chemical reaction. All QM/MM-ONIOM calculations were performed using the Gaussian 16 Revision C.01 program package [18]. The geometries of the PurD complex with substrates for the initial and final states of the reaction were determined using the QM/MM-ONIOM method, which uses microiterations for the optimization.

### 2.3. MEP Calculation

An MEP connecting the initial and final states of the reaction was determined using the string method [19,20] which has been used in various studies to determine a representative reaction path of the chemical processes, including enzyme reactions [15,16]. According to the string method, first, we prepare a string of states, called images, and move each image in the direction of the force derived from a potential energy gradient. Then, we reparametrize the string to enforce approximately equal arc lengths between neighboring images. The MEP was determined by iteratively repeating this process.

In this study, we determined the MEP connecting the reactant and product states of the substrates by relaxing the enzyme structure. PurD enzyme catalyzes the chemical recombination of all atoms in the substrates involved in the QM part of the QM/MM model, where the conformation of the enzyme involved in the MM part can change during the reaction. However, its chemical structure remains unchanged. Thus, each path optimization was carried out as follows. First, the QM part was evolved according to the string method by fixing the MM part, and then the MM part was relaxed by fixing the QM part.

The string method, combined with the QM/MM-ONIOM method, was carried out using an in-house program. The technique for finding an MEP using the string method can be briefly summarized as follows [19,20]. Let V denote a potential energy of the system, and let ζ be a curve connecting points **A** and **B** in configuration space. If curve $\zeta^{*}$ is an MEP, the potential energy gradient ∇V perpendicular to $\zeta^{*}$ is zero everywhere along the curve, which satisfies

(1) $${\left(\nabla V\right)}^{⊥}\left(\zeta^{*}\right)=0.$$

Let $\hat{P}$ denote a projector on a plane perpendicular to the curve:

(2) $$\hat{P}=\hat{I}-\hat{\tau }\cdot {\hat{\tau }}^{T},$$

where $\hat{I}$ is the unit matrix and $\hat{\tau }$ is a normalized tangent vector to the curve. Equation (1) can be rewritten as

(3) $$\hat{P}\;\nabla V\left(\zeta^{*}\right)=0.$$

Let an arbitrary curve ζ be represented by a number of images as

(4) ζ={z(σ): σ∈[0, 1]},

where z(0)=A and z(1)=B. A sequence of images defines a *string*. The reaction coordinate parameter *σ* ∈ [0,1] was used to parameterize MEPs. The basic idea of the string method [19,20] is to find an MEP by evolving the images according to the potential force at that point:

(5) $$\dot{z}\left(\sigma \right)=-\nabla V\left(z\left(\sigma \right)\right),$$

where $\dot{z}$ denotes the time derivative of z. Equation (5) can be integrated with respect to time using any suitable ordinary differential equation (ODE) solver. If the forward Euler method is used, the new image after an ODE step is given by

(6) $$z^{⋆}\left(\sigma \right)=z\left(\sigma \right)-\nabla V\left(z\left(\sigma \right)\right)\;\Delta t,$$

where Δt is the size of each ODE step. After a certain number of evolution steps, the images are redistributed along the string to enforce equal arc lengths between adjacent images, which are needed to avoid image clustering.

An initial path was prepared by linearly interpolating the molecular geometries of the reactant and product states of the substrates in Cartesian coordinates with 16 discrete points. The update step size in the path optimization was set to 1.0 Bohr. The convergence of the path optimization was examined using the average value of the potential energy change while updating the path Δ*V*. After 514 path-optimization cycles, Δ*V* converged at a value less than 1.0 × 10^−10^ Hartree. The resultant MEP was linearly interpolated with a total of 45 discrete points between the two end points in the Cartesian coordinate space to improve the accuracy of the free energy calculations.

### 2.4. Free-Energy Calculation

Free energy is a fundamental energetic property of chemical reactions. The free-energy change along a reaction coordinate, called the free-energy profile, is helpful for understanding the reaction mechanism. Many approaches for calculating free-energy changes based on the QM/MM method have been proposed. In this study, the free-energy changes along the MEP were examined using a perturbation method combined with the QM/MM model [21,22]. Recently, one of the authors of this paper applied the QM/MM free-energy perturbation (QM/MM-FEP) method to analyze the free-energy profile for chemical reactions of excited state molecules in aggregates [23,24]. From the QM/MM-FEP theory, the free-energy difference between two adjoining states, *A* and *B*, in a QM/MM system can be defined as

(7) $$\Delta F_{\mathrm{qm}/\mathrm{mm}}{}^{\left(A\to B\right)}=\Delta E_{\mathrm{qm}}^{\left(A\to B\right)}+\Delta F_{\mathrm{int}}^{\left(A\to B\right)}.$$

The $\Delta E_{\mathrm{qm}}^{\left(A\to B\right)}$ term is given by

(8) $$\Delta E_{\mathrm{qm}}^{\left(A\to B\right)}=\langle E_{\mathrm{qm}}{(r^{\left(B\right)},R^{\left(B\right)})\rangle }_{R^{\left(B\right)}}-\langle E_{\mathrm{qm}}{(r^{\left(A\right)},R^{\left(A\right)})\rangle }_{R^{\left(A\right)}}$$

where ${\langle \cdots \rangle }_{R^{\left(A\right)}}$ represents the ensemble average over the MM subsystem at the *A*-th state. Here, the perturbation corresponds to the forward or backward movement of the QM atoms while fixing all MM atoms. We performed canonical molecular dynamics (MD) simulations by fixing the QM atoms and obtained the required ensembles. The QM/MM single-point calculations were performed using the ONIOM method [15,16]. The $\Delta F_{\mathrm{int}}^{\left(A\to B\right)}$ term is related to the average of the function of the energy difference between the *A-*th and *B*-th states evaluated by sampling for the *A*-th state:

(9) $$\Delta F_{\mathrm{int}}^{\left(A\to B\right)}=-\frac{1}{\beta }\ln\langle \exp{[-\beta \Delta E_{\mathrm{int}}^{\left(A\to B\right)}]\rangle }_{R^{\left(A\right)}},$$

where *β* is a reciprocal temperature, and

(10) $$\Delta E_{\mathrm{int}}^{\left(A\to B\right)}=E_{\mathrm{int}}\left(r^{\left(B\right)},R^{\left(A\right)};\Psi^{\left(B\right)}\right)-E_{\mathrm{int}}\left(r^{\left(A\right)},R^{\left(A\right)};\Psi^{\left(A\right)}\right).$$

The first term of Equation (10), $E_{\mathrm{int}}\left(r^{\left(B\right)},R^{\left(A\right)};\Psi^{\left(B\right)}\right),\;$ represents the perturbation corresponding to a conformational change of the QM solute molecules from $r^{\left(A\right)}$ to $r^{\left(B\right)}$, accompanied by a change in the electronic wavefunction from $\Psi^{\left(A\right)}$ to $\Psi^{\left(B\right)}$, when all MM solvent molecules are fixed at $R^{\left(A\right)}$. The $E_{\mathrm{int}}$ term consists of additive contributions originating from van der Waals and electrostatic interactions:

(11) $$E_{\mathrm{int}}\left(r^{\left(B\right)},R^{\left(A\right)};\Psi^{\left(B\right)}\right)=E_{\mathrm{vdW}}\left(r^{\left(B\right)},R^{\left(A\right)}\right)+E_{\mathrm{es}}\left(r^{\left(B\right)},R^{\left(A\right)};\Psi^{\left(B\right)}\right).$$

Here, the van der Waals interaction can be described with the pairwise Lennard-Jones potential function:

(12) $$E_{\mathrm{vdW}}\left(r^{\left(B\right)},R^{\left(A\right)}\right)=\underset{\alpha \in \mathrm{QM}}{\sum }\underset{\beta \in \mathrm{MM}}{\sum }4\epsilon_{\alpha \beta }\left[{\left(\frac{\sigma_{\alpha \beta }}{\left|r_{\alpha }^{\left(B\right)}-R_{\beta }^{\left(A\right)}\right|}\right)}^{12}-{\left(\frac{\sigma_{\alpha \beta }}{\left|r_{\alpha }^{\left(B\right)}-R_{\beta }^{\left(A\right)}\right|}\right)}^{6}\right]$$

With the parameters of $\epsilon_{\alpha \beta }$ and $\sigma_{\alpha \beta }$ between the *α*-th QM and *β*-th MM atoms, the electrostatic interaction can be approximately expressed by an effective classical representation as follows:

(13) $$E_{\mathrm{es}}\left(r^{\left(B\right)},R^{\left(A\right)};\Psi^{\left(B\right)}\right)=\underset{\alpha \in \mathrm{QM}}{\sum }\underset{\beta \in \mathrm{MM}}{\sum }\frac{Q_{\alpha }^{\left(B\right)}q_{\beta }}{\left|r_{\alpha }^{\left(B\right)}-R_{\beta }^{\left(A\right)}\right|},$$

where $Q_{\alpha }^{\left(B\right)}$ is the point charge on the *α*-th QM atom in the solute molecule determined at the *B*-th state on the MEP, which can be determined by the electrostatic potential fitting method according to the Hu-Lu-Yang scheme [25] based on the electronic wavefunction of $\Psi^{\left(B\right)}$, and *q* is the classical charge on the *β*-th MM atom in enzyme and solvent molecules, which were determined from the parameter sets given in the Amber force field. The QM/MM-FEP calculations were performed using an in-house developed program.

### 2.5. MD Simulations

Free-energy calculation based on the QM/MM-FEP method requires ensemble averages over the MM subsystem while fixing all QM atoms. In this study, canonical MD simulations of PurD in an explicit water box were performed to obtain the necessary ensembles at 358 K, which is the optimal temperature for *Aquifex aeolicus*, a thermophilic bacterium. All MD simulations were performed using the AMBER 18 software package [26,27]. The MM subsystem was modeled using the ff14SB variant of the AMBER force field [28]. The system was solvated with a water shell of 8 Å around the protein. The TIP3P water model was used to describe the solvent [29]. Four sodium cation atoms were added to neutralize the system. The system includes 32,108 atoms in total.

MD simulations were performed for 45 QM/MM systems separately, where QM molecules were fixed to optimized structure on the MEP, and MM molecules were thermally fluctuated according to the given canonical ensembles. The geometry of each system was optimized using the steepest descent algorithm for 500 steps, followed by the conjugate gradient algorithm for 4500 steps. After geometry optimization, each system was heated until the temperature (*T*) reached 358 K over a period of 200 ps in the constant volume and temperature (*NVT*) ensemble while applying a harmonic restraint of 2 kcal mol^−1^ Å^−2^ on the system, except for the hydrogen atoms. The temperature was regulated using the weak-coupling algorithm. After heating, 200 ps of MD simulations were performed to equilibrate the system in the *NPT* ensemble at *T* = 358 K and a pressure (*P*) of 1.0 atm. The pressure was maintained using a Berendsen barostat. After equilibration, additional 7-ns MD simulations were performed in the *NPT* ensemble at *T* = 358 K and *P* = 1.0 atm. During MD simulations, all covalent bond lengths were constrained using the SHAKE algorithm [30]. The time step of MD simulations was set to 2 fs. A cutoff for the non-bonded intermolecular interactions was set to 8 Å. Long-range electrostatic interactions were treated using the particle-mesh Ewald method [31]. Finally, 45 MD simulations were performed for 3 ns in the *NPT* ensemble at *T* = 358 K and *P* = 1.0 atm with fixing QM molecules to the optimized structure at each point on the MEP. The 300 samples used for the QM/MM-FEP analysis were obtained from the 3-ns MD trajectory at each discrete point on the MEP.

## 3. Results and Discussion

### 3.1. Optimized Structures

The first step of the GAR synthesis reaction catalyzed by PurD to generate glycyl-phosphate, as shown in Figure 1, is accompanied by the following structural changes. In the initial state of the reaction, ATP, two Mg^2+^ ions, and glycine bind to PurD. During the reaction, one of the three phosphoric groups in ATP is cleaved and transferred to glycine upon hydrolysis, which yields ADP and glycyl-phosphate at the final state. At present, the structure of the glycyl-phosphate intermediate bound to PurD has not been experimentally determined. In this study, we have determined the optimal geometries for the initial and final conformational states of the reactions based on the QM/MM-ONIOM model.

**Figure 2:**
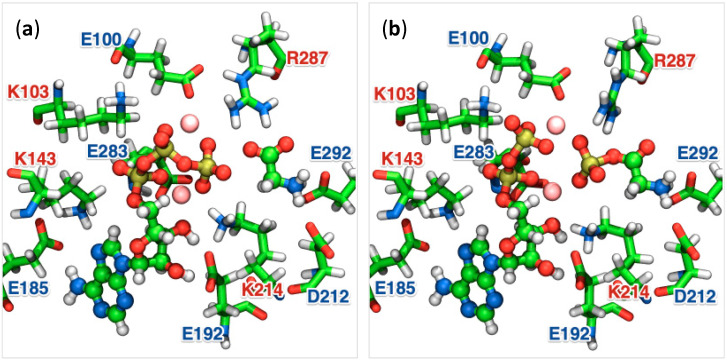
Reaction sites on the optimized structures of PurD in the (**a**) initial and (**b**) final states of the reaction that produces glycyl-phosphate from glycine and ATP, corresponding to Reaction 1 in Figure 1. Substrates involved in the reaction are represented by a ball-and-stick model and each element is represented by a different colored ball. Charged amino acid residues that interact with the substrate are represented by a licorice model.

Figure 2 shows the substrate-binding site of the optimized geometries at the initial and final states of the reaction, which demonstrate the conformational changes of substrates accompanied by the conformational changes of amino acid residues adjacent to them within 2 Å. Sixteen amino acid residues are in the vicinity of the reaction center within the range of 2 Å from the substrate molecules, of which 11 amino acid residues, that is, 69% of the total, were charged residues. PurD contains 423 amino acid residues, of which 134 (32%) are charged amino acid residues, indicating that a high percentage of charged amino acid residues are localized in the substrate-binding site. Binding of the substrate molecules to the 10 charged amino acid residues within the ATP-grasp domain of PurD is as follows: ATP was recognized by Lys103, Lys143, Glu185, Glu192, and Lys214; a glycine molecule was bound to Asp212, Arg287, and Glu292; two Mg^2+^ ions were recognized by Glu100 and Lys214. Among the 10 charged amino acid residues that were bound to the substrate molecules, 6 were negatively charged and 4 were positively charged, indicating that there were more negatively charged residues at the substrate-binding site. As shown in Figure 2, two Mg^2+^ ions maintained coordination bonds with the oxygen atoms of ATP in both the initial and final states of the reaction, where one of the two binds at the β- and γ-positions, and the other at the α- and γ-positions.

### 3.2. MEP

**Figure 3:**
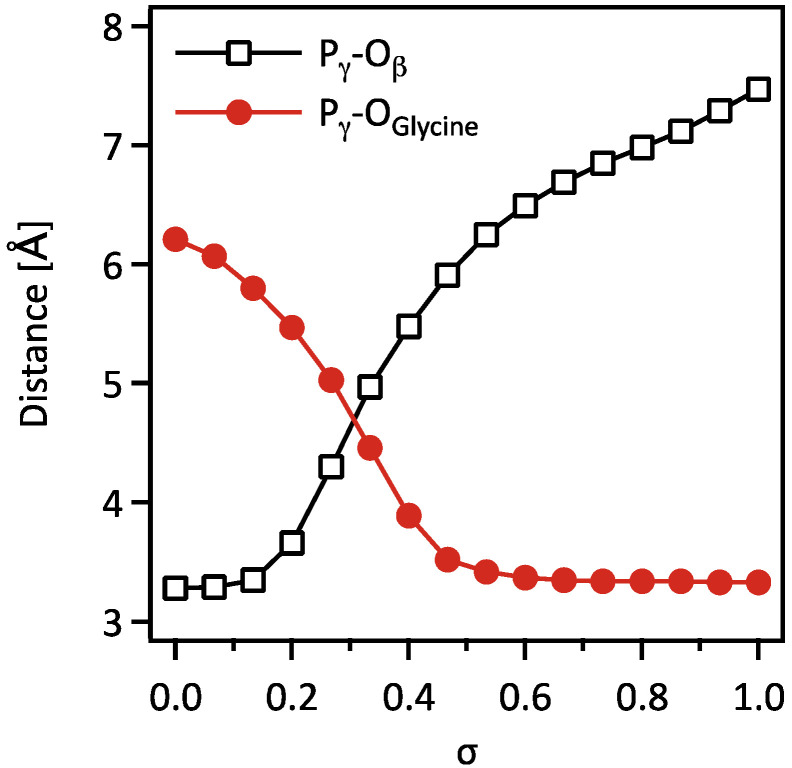
Changes in the interatomic distances between the phosphorus (P) and oxygen (O) atoms at the γ- and β-positions of ATP, P_γ_-O_β_, and at the carbonyl group of glycine, P_γ_-O_glycine_, along the minimum energy path.

**Figure 4:**
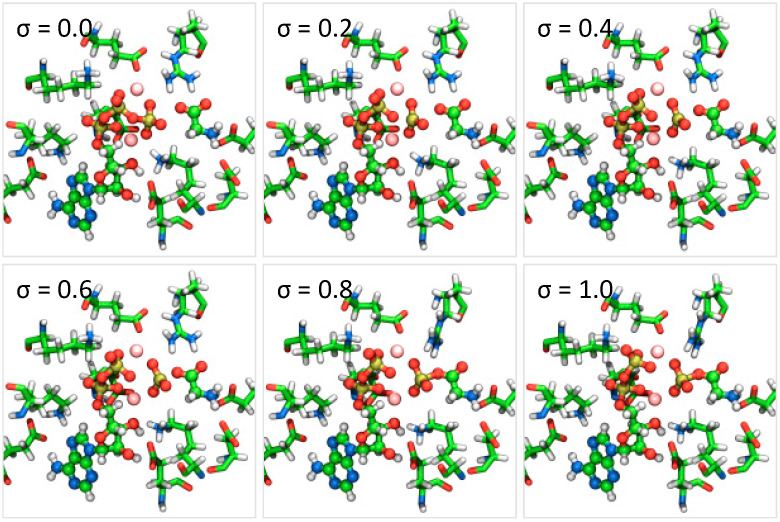
Structural changes of the substrates and its neighboring amino acid residues of PurD at six points (σ = 0.0–1.0) on the minimum energy path. The substrates involved in the reaction are represented by a ball-and-stick model and each element is represented by a different colored ball. Charged amino acid residues that interact with the substrate are represented by a licorice model.

The MEP of the catalytic reaction in PurD was determined using the string method combined with the QM/MM approach, which connects the initial and final states, as shown in Figure 2. Figure 3 shows the changes in the interatomic distance between the phosphorus and oxygen atoms at the γ- and β-positions of ATP, P_γ_-O_β_, and the distance between the phosphorus and oxygen atoms at the carbonyl group of glycine, P_γ_-O_glycine_. The reaction coordinate parameter *σ* represents the degree of conformational changes of the substrate molecules (ATP^4−^, two Mg^2+^ ions, and glycine) along the MEP, which is normalized to zero at the initial state and one at the final state. In Figure 3, the distances were plotted against *σ*, which gives a detailed account of the structural changes during the reaction, indicating the hydrolysis of the γ-phosphate of ATP and the phosphorylation of glycine. Figure 4 shows the conformational changes of the substrates and its neighboring amino acid residues of PurD at six points (σ = 0.0–1.0) on the MEP, where a phosphoryl transfers from ATP to an adjacent glycine, yielding a glycyl-phosphate and ADP.

As shown in Figure 3, at the initial state of the reaction (*σ* = 0.0 to 0.1), the P_γ_-O_β_ distance remained unchanged, and only the P_γ_-O_glycine_ distance decreased, as glycine approaches ATP before the ATP hydrolysis. As shown in Figure 3 and Figure 4, a phosphoryl group dissociates from ATP and forms a planar structure at the reaction coordinate of *σ* = 0.2, and the P_γ_-O_glycine_ distance reaches 3.37 Å at *σ* = 0.6, which indicates the production of glycyl-phosphate. At the late stage of the reaction (*σ* = 0.6 to 1.0), the P_γ_-O_glycine_ distance remained unchanged. However, the P_γ_-O_β_ distance continued to change and increased by nearly 1 Å towards the completion of the reaction.

### 3.3. Free-Energy Profile

**Figure 5:**
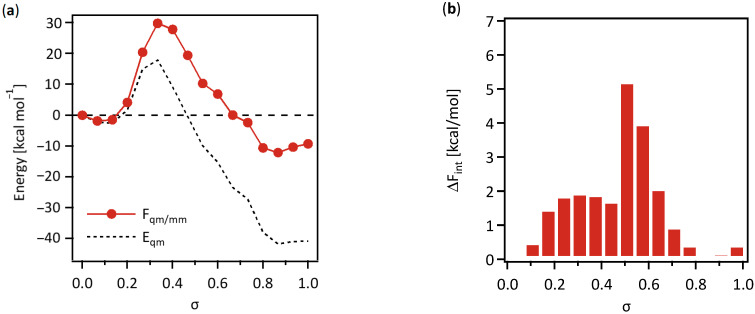
(**a**) Change in the free-energy along the MEP relative to that in the QM energy. (**b**) Change in the corresponding QM/MM free-energy contribution.

The free-energy change along the MEP was determined using the QM/MM-FEP method, which is given as the sum of the QM energies and free-energy contributions ascribed to the QM/MM interactions. Figure 5a shows the change in the free-energy along the MEP relative to that in the QM energy. Figure 5b shows the corresponding QM/MM free-energy contribution.

As shown in Figure 5a, the free-energy profile shows that the reaction requires an activation energy of 26.1 kcal mol^−1^, and the total change in the free-energy reaches −17.0 kcal mol^−1^ at the end of the reaction. The total free-energy change indicates that the reaction is exergonic and can occur spontaneously in the enzyme.

The activation energy of 26.1 kcal mol^−1^ is large compared to typical enzymatic reactions [32], even if we consider the fact that *Aquifex aeolicus* is hyperthermophilic. In the free-energy calculation using the QM/MM-FEP method, MD simulations were performed by fixing all the substrates treated as the QM subsystem to obtain necessary ensemble averages over the MM subsystem. In the current enzymatic reaction, the phosphoryl group moves between two spatially distant substrate molecules. In such a case, the activation energy can tend to be overestimated when the free-energy change is computed using the QM/MM-FEP method because it is not flexible enough to consider the structural fluctuations of the substrate molecules in the QM region.

The change in the QM energy along the MEP is attributed to the rearrangement of atoms in the substrate molecules during the reaction, where interaction with the neighboring amino acid residues involved in the MM-model moiety was considered in the QM/MM Hamiltonian as the electrostatic interaction with the embedded charges of atoms in the enzyme. The profile of the QM energy has a maximum value of 17.9 kcal mol^−1^ at *σ* = 0.33, which is corresponding to the transition state shown in the free-energy profile and reaches −40.8 kcal mol^−1^ at the end. The large difference in quantity from the free-energy profile indicates the importance of the free-energy contribution.

Changes in the intermolecular interactions between the QM- and MM-model moieties along the MEP is shown in Figure 5b as the QM/MM free-energy contribution, Δ*F*_int_. The QM/MM free-energy contributions are positively large around the transition state, forming an intermediate complex of ADP-PO_3_-glycine at *σ* = 0.33, where the value reaches 1.93 kcal mol^−1^ corresponding to the QM/MM free-energy difference between the points at *σ* = 0.3 on the MEP. The largest value of Δ*F*_int_ is 5.2 kcal mol^−1^ at *σ* = 0.5 on the MEP, where the phosphoryl group is about to reach glycine to produce the glycyl-phosphate. The evident change in the QM/MM free-energy contribution shown in Figure 5b indicates a notable change in the intermolecular interactions between the substrate molecules and the enzyme. Therefore, the next step was to conduct a more detailed analysis to clarify the cause of the marked changes in the interaction between the enzyme and substrate molecules during this state of the reaction.

### 3.4. Partial Atomic Charges

**Figure 6:**
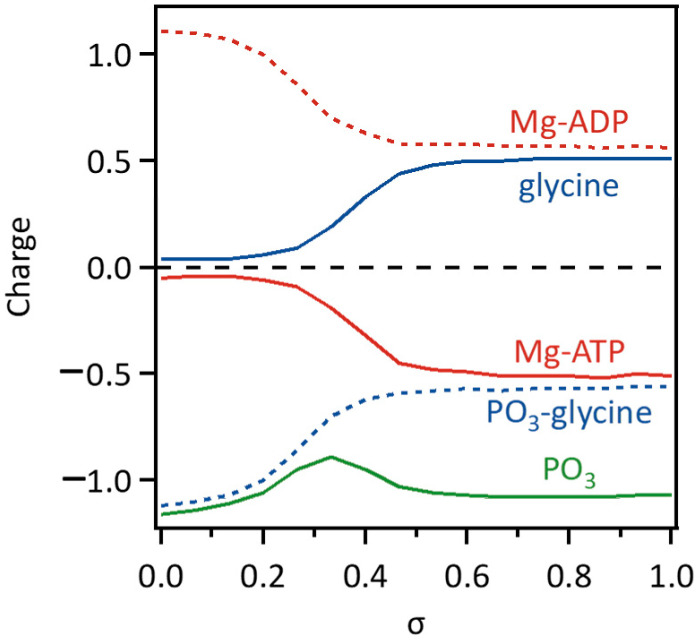
Changes in the arithmetic sums of the partial atomic charges (*Q*) for five atomic groups of Mg-ATP, consisting of two Mg^2+^ ions and ATP^4−^, glycine, phosphate (PO_3_), and Mg-ADP, consisting of two Mg^2+^ ions and ADP^3−^, and glycyl-phosphate (PO_3_-Gly).

**Figure 7:**
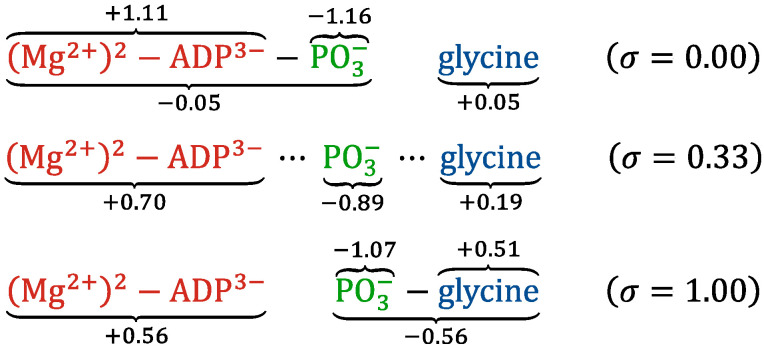
Summary of the re-distribution of the partial atomic charges of the ligand molecules described in Figure 6.

The rearrangement of chemical bonds among the substrate molecules along the MEP can change the distribution of partial atomic charges. Figure 6 shows the changes in the arithmetic sums of the partial charges for five atomic groups of Mg-ATP, consisting of two Mg^2+^ ions and ATP^4−^, glycine, phosphate (PO_3_^−^), and Mg-ADP, consisting of two Mg^2+^ ions and ADP^3−^, and glycyl-phosphate (Gly-PO_3_). Figure 7 shows a summary of the redistribution of the partial atomic charges of the ligand molecules. Here, each partial charge on a substrate was determined using the Hu-Lu-Yang fitting method [25] applied to the wavefunction calculated at the B3LYP/6-31G(d,p) level of theory, where each geometry of the substrate was extracted from the images of the MEP determined using the QM/MM-ONIOM string method.

As shown in Figure 6, the overall tendency of the distribution of partial atomic charges clearly changed from *σ* = 0.2 to 0.5. The glycine moiety is almost neutral at the initial state of the reaction; after *σ* = 0.2, the closer the phosphate gets to the glycine, the larger its increase in the partial charge; then it reached +0.50 at *σ* = 0.6, where glycyl-phosphate was produced. The calculated partial charge of the glycyl-phosphate moiety was −0.56 at the end of the reaction, although the formal charge of glycyl-phosphate was −1. The Mg-ATP moiety was also almost neutral initially; however, after *σ* = 0.2, its partial charge decreased and reached −0.49 at *σ* = 0.6, where ATP was hydrolyzed. The calculated partial charge of the Mg-ADP moiety was +0.56 at the end, although the formal charge was +1. The partial charge of the phosphate moiety was −1.16 at the initial state, and its value, −1.07, at the end almost remained unchanged. However, it showed a small peak of −0.89 at *σ* = 0.33 during the reaction, where the partial charges of the Mg-ADP and glycine moieties were +0.70 and +0.19, respectively.

The redistribution of partial atomic charges on the substrate molecules (*Q*) affects the electrostatic interactions with the enzyme (*E*_es_), which contributes significantly to the QM/MM free-energy change, Δ*F*_int_, as described in Equations (9) and (13). As shown in Figure 5b, in the region of *σ* = 0.2–0.5, where the partial atomic charges on the substrate molecules changed overall, the value of Δ*F*_int_ increased remarkably, indicating that the electrostatic repulsion between the substrate molecules and the enzyme increased because of the redistribution of partial atomic charges associated with the reaction process. As shown in Figure 7, both the glycine and Mg-ATP moieties were neutral at the beginning of the reaction. However, glycyl-phosphate was negatively charged (−0.56) and the Mg-ADP moiety was positively charged (+0.56) at the end of the reaction, where the formal charges of glycyl-phosphate and the Mg-ADP moiety were −1 and +1, respectively. This indicates that the partial atomic charges were re-distributed to delocalize within two sites of the glycyl-phosphate and Mg-ADP moieties at the end of the reaction, instead of localizing according to the formal charges. The delocalization of the atomic partial charges might result from the fact that the negatively charged PO_3_^−^ moiety remains coordinated to two Mg^2+^ cations throughout the reaction process, as shown in Figure 4. If the partial atomic charges of the substrate molecules are localized to the two sites according to the formal charges, the electrostatic repulsion with the surrounding charged amino acid residues may become too strong for the enzymatic reaction to proceed. The results of the free-energy profile analysis indicate that the two Mg^2+^ ions bound to PurD can play an important role in the progression of the enzymatic reaction by appropriately adjusting the partial atomic charges on the substrate molecules.

## 4. Conclusions

In this study, we investigated the reaction mechanism of the second step in the de novo biosynthetic pathway of purine nucleotides catalyzed by PurD enzyme based on the free-energy profile analysis. An efficient computational protocol for analyzing the free-energy profile of the glycine phosphorylation process catalyzed by PurD was developed, which examines the free-energy change along an MEP based on a perturbation method combined with the QM/MM hybrid model. The energetics calculated from the MEP provided valuable information for a comprehensive understanding of the reaction process in PurD.

The free-energy profile revealed that the phosphorylation of glycine by ATP in PurD requires an activation energy of 26.1 kcal mol^−1^, and the total change in the free-energy reaches −17.0 kcal mol^−1^ at the end of the reaction. In this reaction process, the change in the QM/MM free-energy contribution was remarkable, indicating a notable change in the intermolecular interactions between the substrate molecules and the enzyme.

Further detailed analysis of the changes in the partial atomic charges of the substrate molecules revealed that the electrostatic intermolecular interactions between the substrate molecules and the charged amino acid residues in the ATP-grasp domain of PurD play an important role in the reaction process. Of particular interest, the partial atomic charges of the substrate molecules were re-distributed to delocalize within two sites of glycyl-phosphate and Mg-ADP moieties at the end of the reaction, instead of localizing according to the formal charges. This suggests that the two Mg^2+^ ions bound to PurD play an important role in the progression of the enzymatic reaction by appropriately adjusting the partial atomic charges of the substrate molecules.

The free-energy profile analysis of PurD is a starting point for understanding the functional relationships among the ATP-grasp superfamily enzymes. The computational protocol discussed in this study can be used to elucidate the detailed reaction mechanism of the other ATP-grasp superfamily enzymes, which provides molecular-level insight into the evolutionary process of the de novo biosynthetic pathway of purine nucleotides.

## Data Availability

The raw data presented in this study are available here: https://github.com/yamnor/PurD (accessed on 8 February 2022).
